# Relationship between depression and sex steroid hormone among women with epilepsy

**DOI:** 10.3389/fnins.2024.1370533

**Published:** 2024-04-22

**Authors:** Luqman Ogunjimi, Akinyinka Alabi, Ibironke Oyenuga, Jeremiah Ogunkunle, Emmanuel Kasumu, Oluwaseun Ogunsanya, Oluwatobiloba Oluseyije, Pelumi Ogunbayo, Omorojo Idume, Adeola Kasali, Sarah Adesi, Mariam Oyebowale, Damilola Ogungbemi, Aderonke Aderinola, Emmanuel Irokosu, Abdullahi Murtala, Bamidele Osalusi

**Affiliations:** ^1^Department of Pharmacology and Therapeutics, Obafemi Awolowo College of Health Sciences, Olabisi Onabanjo University, Sagamu, Ogun State, Nigeria; ^2^Department of Biochemistry, Obafemi Awolowo College of Health Sciences, Olabisi Onabanjo University, Sagamu, Ogun State, Nigeria; ^3^Department of Medicine, Obafemi Awolowo College of Health Sciences, Olabisi Onabanjo University, Sagamu, Ogun State, Nigeria

**Keywords:** depression, sex steroid hormones, anti-seizure medication, epilepsy, women

## Abstract

**Introduction:**

Sex steroid hormones are emerging significant biomarkers of depression among Women with Epilepsy (WWE) with promising prognostic potential and therapeutic end point. Therefore, the study is aimed at exploring the association between sex steroids hormones, Anti-seizure Medication (ASM) and depression among WWE.

**Methodology:**

A baseline questionnaire was used to obtain socio-demographics and clinical characteristic from one hundred and twelve (112) WWE and 50 age matched healthy control. The diagnosis of epilepsy and Electroencephalography (EEG) description was based on 2017 International League Against Epilepsy (ILAE) criteria. Blood samples were collected from cases and control during Luteal Phase (LP) and Follicular Phase (FP). The Zung Self-Rating Depression Scale (ZSRDS) was used to assess depression.

**Result:**

The prevalence of depression among WWE is 18.8%, with a significant difference between the level of formal education (p0.000), age (p0.000), and mean ZSRDS (p0.000) among cases and control. There is a statistical difference in hormonal levels between cases and control with regards to higher testosterone [3.28 ± 9.99 vs. 0.31 ± 0.30; p0.037], lower FP prolactin [16.37 ± 20.14 vs. 17.20 ± 7.44; p0.778], and lower LP prolactin [15.74 ± 18.22 vs. 17.67 ± 7.27; p0.473]. Testosterone (p0.024), FP Follicle Stimulating Hormone (FSH) (p0.009), FP Estradiol (p0.006), LP FSH (p0.031), LP Progesterone (p0.023), and LP Prolactin (p0.000) were associated with depression. However, only prolactin (p0.042) and testosterone (p0.000) predicts depression among WWE.

**Conclusion:**

There was higher mean depression score, lower prolactin and higher testosterone level among cases compared to control. Furthermore, there was lower prolactin and higher testosterone level in Carbamazepine (CBZ) group compared to Levetiracetam (LEV) groups.

## 1 Background

The International League Against Epilepsy (ILAE) defined epilepsy as a disease with either recurrent unprovoked seizures (i.e., two or more unprovoked seizures occurring at least 24 h apart) or a heightened tendency toward recurrent unprovoked seizures. Depression is about two to three times more common in people with epilepsy (PWE). Increasing evidence reveals that epilepsy and depression may share common pathophysiological, genetic, and environmental mechanisms (Kanner et al., 2012; Ogundare et al., 2020). More recently, by using the Zung Self-Reported Depression Scale (ZSRDS), depression was found in 26.9% of epileptic patients compared with 9.7% of controls (Okubadejo et al., 2007). Women with epilepsy (WWE) **face unique** challenges throughout their lives owing to the effects of seizures and anti-seizure medications (ASMs) (Harden and Pennell, 2013; Harden, 2015). The rate of suicide in PWE is approximately 25 times greater than that of the general population (Okubadejo et al., 2007; Coppola et al., 2019).

According to a warning by the Food and Drug Administration (FDA) in 2008, ASMs can increase the risk of suicidal behaviors and ideation. It is unclear whether this increased the risk of suicidal behaviors and whether ideation results from the ASM or the epilepsy itself. Depression can directly increase seizure frequency through the mechanism of sleep deprivation; failure to recognize depression or inadequate treatment can lead to suicide (Cavanna et al., 2009). Some ASMs, such as phenobarbital, topiramate, vigabatrin, tiagabine, gabapentin, levetiracetam (LEV), and zonisamide, may have negative effects on mood. Risk factors for ASM-induced depression include a family history of mood disorders, anxiety, or alcoholism; severe epilepsy; rapid titration; and polytherapy (De Ryck et al., 2014). The functions of the hypothalamic–pituitary axis, including the production of luteinizing hormone (LH), follicle stimulating hormone (FSH), gonadotropin-releasing hormone (GnRH), prolactin (PRL), estrogen, testosterone, and dehydroepiandrosterone (DHEAS), are modified in many WWE (Harden and Pennell, 2013; Herzog, 2015; Hamson et al., 2016). Understanding the differential effects of different sex steroid hormones remains a critical step leading to more effective therapies that are most likely to impact on the overall quality of life in WWE (Harden, 2015; Shiono et al., 2019). In addition, depression remains one of the huge burden of epilepsy and is even more common in WWE since women are twice likely as men to suffer from depression, and the female gender could be considered a major risk factor for developing this condition. Emerging evidence suggests that cognitive effects are influenced by specific hormone formulations and that progesterone is more likely to be associated with positive outcomes than its synthetic counterparts (Verrotti et al., 2009; Shiono et al., 2019). Therefore, this study aimed to determine the relationship between sex steroid hormones, ASM, and depression in WWE.

## 2 Methodology

This is an observational case–control study comprising 112 WWE aged 15 years and above, which was later sub-grouped into carbamazepine (CBZ) and LEV groups based on the ASM they are on, and fifty (50) healthy age-matched controls. This was carried out after due ethical clearance has been obtained from Health Research Ethics Committee (HREC) of Olabisi Onabanjo University Teaching Hospital (OOUTH). Sagamu with assigned number OOUTH/HREC/275/2019AP and Federal Medical Center, Abeokuta (FMCA) with assigned number FMCA/243/HREC/03/2016/15. Sample size was estimated using the convenience sampling method. Epilepsy was confirmed with electroencephalography (EEG) and classified according to the ILAE classification. Patients with primary/secondary hypogonadism, amenorrhea >6 weeks, background psychiatric disorder, on steroids/oral/injectable contraception, pregnancy, and history of previous or ongoing endocrinopathies were excluded. Participants with no background history of seizure disorder were grouped as controls. The epilepsy registry at the FMCA and OOUTH Sagamu was used to record all cases of epilepsy reported during the period of study, irrespective of the outcome. A baseline questionnaire was used to obtain information from the study participants, including socio-demographic characteristics (sex, age, educational level, living condition, and marital status) and clinical history relating to epilepsy (age of onset, types of seizure, aura, precipitant, ASM, and ZSRDS score). Information regarding weight, height, pulse, blood pressure, and past and current medications was recorded. The International 10–20 system of EEG electrode placement was carried out on all participants using a Phoenix digital 16-channel EEG machine. This recording was done at presentation for all participants, and each recording took approximately 20–30 min. The reports were interpreted by a neurologist, while an artifactual result was repeated. The ZSRDS was used to assess the mood of cases and controls (Xu et al., 2004; Romera et al., 2008; Jokelainen et al., 2019). The ZSRDS is a 20-item question scale that rates the four common characteristics of depression: the pervasive effect, the physiological equivalents, other disturbances, and psychomotor activities. There are ten positively worded and ten negatively worded questions. Each question was scored on a scale of 1–4 (a little of the time, some of the time, good part of the time, and most of the time). The total score was derived by summing the individual item scores. The scores ranges from 20 to 80, with a score <50 indicating not depressed and a score >50 indicating depressed. Ten milliliters of venous blood samples was drawn aseptically from each subject twice, and this was subsequently dispensed into lithium heparin bottles. The first sample was taken during the ovulatory phase on the 10^th^-13^th^ day of the menstrual cycle, while the second sample was taken during the luteal phase on the 21^st^-24^th^ day of the cycle. Ogunjimi et al. (2021) have previously explained the blood sample analysis. All participants who satisfied the inclusion criteria had estrogen (pg/mL), progesterone (ng/mL), PRL (ng/mL), LH (mIU/mL), FSH (mIU/mL), and sex hormone binding globulin (SHBG) (mmol/L) assayed during the two phases of the menstrual cycle: follicular phase (FP) and luteal phase (LP). Data procured from the participants were coded and analyzed using the IBM Statistical Package of Social Sciences (SPSS) Version 23. Socio-demographic and clinical characteristics of study participants were presented in tables in terms of frequency (percentage) using Pearson's chi-square test, except age, which was presented as means ± SD using the independent *t*-test. The Pearson chi-square or Fisher's exact test was used to compare categorical variables between cases and controls, while Student's independent *t-*test or Wilcoxon rank-sum test was used to determine differences between cases and controls in the continuous variables. The independent *t*-test was also used to compare the hormonal level between cases and controls, depressed and controls, not depressed and controls, and depressed and not depressed, whereas analysis of variance (ANOVA) was used to compare the hormonal level with depressed, not depressed, and control. A linear regression model was used to predict depression among epilepsy patients using the hormonal level. A *p*-value <0.05 indicated statistical significance.

## 3 Result

### 3.1 The relationship between socio-demographic characteristics and depressed and not depressed patients

**Figure 1:**
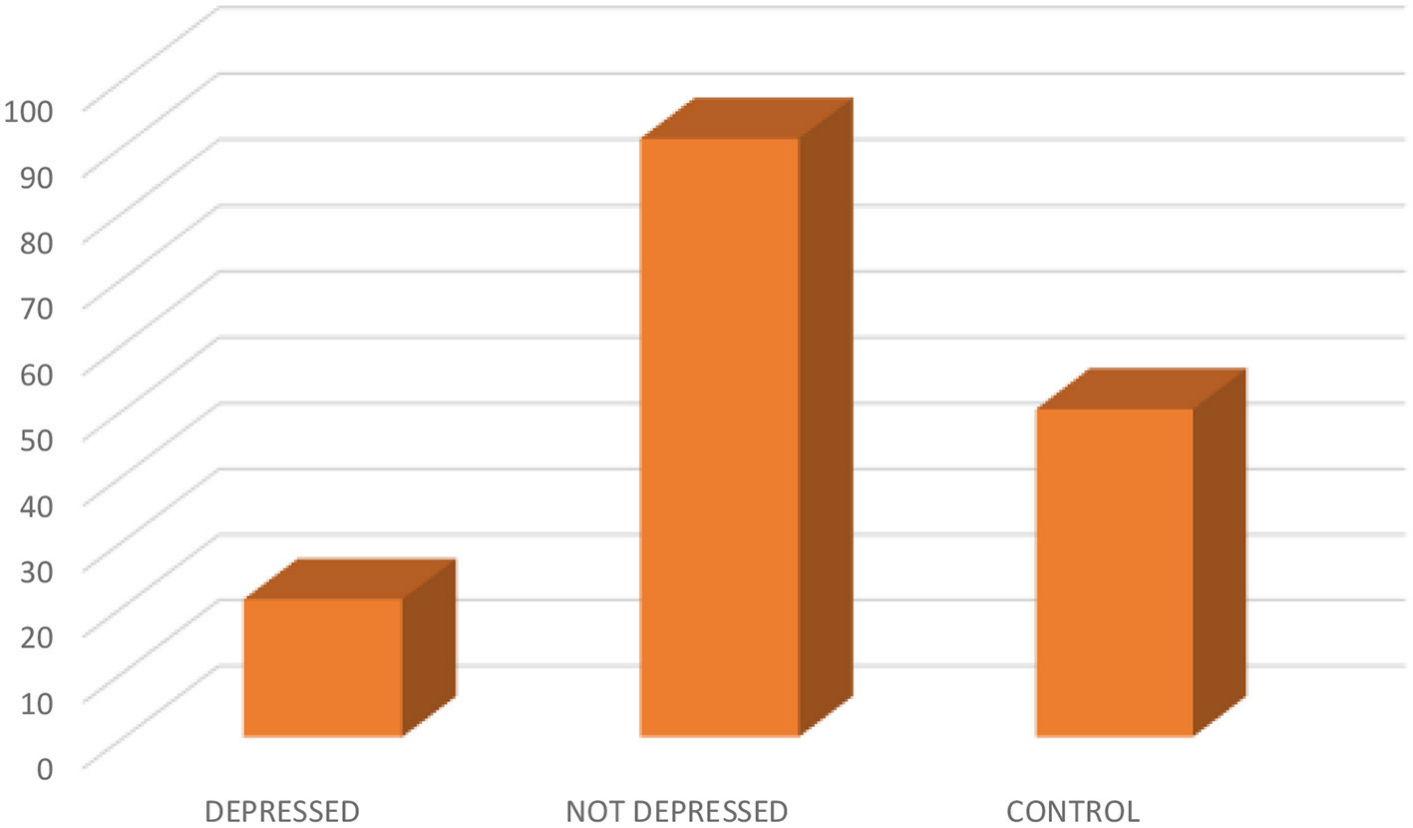
Showing the distribution of depressed, not depressed, and control.

In the comparison of the cases and controls, the socio-demographic characteristics were comparable, except for the educational status (p0.000) (see Table 1A). Out of the 112 epilepsy participants recruited, 21 (18.8) were depressed (see Figure 1). The socio-demographic characteristics were comparable among depressed and not depressed patients, except for handedness (p0.046). Comparing the mean ZSRDS between depressed and not depressed patients, we observed a higher score in the not depressed group, but this group did not attain a statistically significant difference [28.00 ± 11.25 vs. 29.06 ± 7.66; p0.626] (see Table 1B).

**Table 1A:** Showing relationship between socio-demographic characteristics between cases and controls.

**Variables**	**Cases *n =* 112**	**Control *n =* 50**	**Statistical value**	***P***
Age mean	40.11 (19.87)	34.55 (20.58)	t = 1.910	0.058
**Handedness**				
Right	97 (86.6)	48 (96.0)	x^2^ = 3.247	0.072
Left	15 (13.4)	2 (4.0)		
**Ethnicity**				
Hausa	5 (4.5)	0 (0.0)	x^2^ = 3.782	0.151
Igbo	7 (6.2)	1 (2.0)		
Yoruba	100 (89.3)	49 (98.0)		
**Education status**				
Primary	31 (27.7)	0 (0.0)	x^2^ = 65.515	**0.000** ^*^
Secondary	46 (41.1)	0 (0.0)		
Tertiary	35 (31.2)	50 (100.0)		

^*^statistical significance. Bold indicates significant values.

**Table 1B:** Showing predictors of depression patients with epilepsy using linear regression.

**Variables**	**B**	**Std. error**	***p***	**95% CI** **Lower–upper bound**
FP FSH (mIU/mL)	−0.008	0.008	0.303	−0.024–0.008
FP estradiol (pg/mL)	−0.002	0.002	0.344	−0.005–0.002
FP PRL (ng/mL)	−0.029	0.020	0.156	−0.068–0.011
LP progesterone (ng/mL)	0.009	0.006	0.179	−0.004–0.022
LP Estradiol (pg/mL)	0.002	0.001	0.125	−0.001–0.005
LP PRL (ng/mL)	0.038	0.018	**0.042** ^*^	0.001–0.075
Testosterone (ng/dL)	−0.806	0.116	**0.000** ^*^	−1.037 to −0.574

FP, follicular phase; LP, luteal phase; FSH, follicle stimulating hormone; PRL, prolactin; ^*^statistical significance; B, beta; Std, standard; CI, confidence interval. Bold indicates significant values.

### 3.2 Comparison of hormonal level of epilepsy patients and healthy controls

A consistent statistical difference was observed among epilepsy patients and controls with regards to FP FSH [10.48 ± 15.01 vs. 4.56 ± 3.29; p0.007], FP progesterone [7.53 ± 14.20 vs. 15.20 ± 32.18; p0.037], FP estradiol [135.33 ± 143.62 vs. 206.09 ± 77.02; p0.001], LP progesterone [6.92 ± 13.38 vs. 12.08 ± 12.46; p0.022], and testosterone [3.28 ± 9.99 vs. 0.31 ± 0.30; p0.037] (see Table 2).

**Table 2:** Showing the comparison of hormonal level with epilepsy patients and healthy controls.

**Variables**	**Cases *n =* 112 mean ± SD**	**Control** ***n =* 50** **mean ± SD**	***T* value**	***P***
FP FSH (mIU/ml)	10.48 ± 15.01	4.56 ± 3.29	2.754	**0.007** ^*^
FP LH (mIU/ml)	12.77 ± 20.16	7.98 ± 18.35	1.434	0.153
FP progesterone (ng/mL)	7.53 ± 14.20	15.20 ± 32.18	−2.103	**0.037** ^*^
FP estradiol (pg/mL)	135.33 ± 143.62	206.09 ± 77.02	−3.245	**0.001** ^*^
FP PRL (ng/mL)	16.37 ± 20.14	17.20 ± 7.44	−0.282	0.778
LP FSH (mIU/ml)	9.36 ± 12.43	13.20 ± 19.40	−1.509	0.133
LP LH (mIU/ml)	9.21 ± 10.41	7.27 ± 15.40	0.939	0.349
LP progesterone (ng/mL)	6.92 ± 13.38	12.08 ± 12.46	−2.312	**0.022** ^*^
LP estradiol (pg/mL)	176.22 ± 571.61	218.73 ± 103.06	−0.521	0.603
LP PRL (ng/mL)	15.74 ± 18.22	17.67 ± 7.27	−0.720	0.473
Testosterone (ng/dL)	3.28 ± 9.99	0.31 ± 0.30	2.102	**0.037** ^*^

FP, follicular phase; LP, luteal phase; FSH, follicle stimulating hormone; LH, luteinizing hormone; PRL, prolactin. ^*^statistical significance. Bold indicates significant values.

### 3.3 The relationship between the hormonal phase and depression

Comparison of not depressed participants with controls revealed no significant differences in all hormonal levels, whereas comparison of depressed participants and controls revealed higher FP FSH [14.01 ± 19.31 vs. 4.56 ± 3.29; p0.001], higher testosterone [6.15 ± 12.98 vs. 0.31 ± 0.30; p0.024], lower FP estradiol [146.86 ± 162.88 vs. 206.09 ± 77.01; p0.042], lower FP prolactin [10.89 ± 8.09 vs. 17.19 ± 7.44; p0.004], lower LP progesterone [2.97 ± 5.02 vs. 12.08 ± 12.46; p0.002], lower LP estradiol [134.96 ± 147.08 vs. 218.72 ± 103.05; p0.008], and lower LP prolactin [9.81 ± 8.12 vs. 17.66 ± 7.26; p0.000] (see Table 3).

**Table 3:** Showing the relationship between hormonal phases and depression.

**Variables**	**Depressed*N =* 21**	**Not depressed*N =* 90**	**Control*N =* 50**	**F value**	**p1**	**p2**	**p3**
FP FSH (mIU/ml)	14.01 ± 19.31	9.66 ± 13.83	4.56 ± 3.29	4.838	**0.009** ^*^	**0.001** ^*^	0.011
FP LH (mIU/ml)	13.12 ± 12.49	12.69 ± 21.61	7.97 ± 18.35	1.026	0.361	0.245	0.195
FP progesterone (ng/mL)	4.66 ± 13.14	8.20 ± 14.42	15.20 ± 32.18	2.435	0.091	0.153	0.079
FP estradiol (pg/mL)	146.86 ± 162.88	132.64 ± 139.62	206.09 ± 77.01	5.346	**0.006** ^*^	**0.042** ^*^	0.001
FP PRL (ng/mL)	10.89 ± 8.09	17.48 ± 21.66	17.19 ± 7.44	1.152	0.319	**0.004** ^*^	0.927
LP FSH (mIU/ml)	15.71 ± 19.99	7.88 ± 9.46	13.19 ± 19.39	3.562	**0.031** ^*^	0.623	0.031
LP LH (mIU/ml)	13.21 ± 11.39	8.27 ± 10.00	7.26 ± 15.39	1.864	0.159	0.116	0.638
LP progesterone (ng/mL)	2.97 ± 5.02	7.84 ± 14.52	12.08 ± 12.46	3.882	**0.023** ^*^	**0.002** ^*^	0.085
LP estradiol (pg/mL)	134.96 ± 147.08	185.85 ± 631.25	218.72 ± 103.05	0.231	0.794	**0.008** ^*^	0.716
LP PRL (ng/mL)	9.81 ± 8.12	16.95 ± 19.47	17.66 ± 7.26	1.854	0.160	**0.000** ^*^	0.804
Testosterone (ng/dL)	6.15 ± 12.98	2.61 ± 9.12	0.31 ± 0.30	3.802	**0.024** ^*^	**0.002** ^*^	0.076

FP, follicular phase; LP, luteal phase; FSH, follicle stimulating hormone; LH, luteinizing hormone; PRL, prolactin; ^*^statistical significance, ^*^p1 is the *p-*value comparing depressed, not depressed, and control; ^*^p2 is the *p*-value comparing depressed and controls; ^*^p3 is the *p*-value comparing not depressed and controls. Bold indicates significant values.

### 3.4 The relationship between medication and depression

Comparison of the hormonal level of patients on CBZ with LEV revealed no statistical difference in the hormone level, except for LP LH [12.80 ± 13.45 vs. 5.81 ± 4.18; p0.000], which was lower in the LEV group. With regards to the CBZ group and controls, it was deduced that there were higher FP FSH [12.19 ± 16.40 vs. 4.55 ± 3.22; p0.002], higher testosterone [2.42 ± 6.67 vs. 0.31 ± 0.30; p0.027], and lower FP estradiol [127.23 ± 154.93 vs. 206.09 ± 77.01; p0.002].

In addition, comparison of LEV and controls revealed a consistent statistical difference in the hormonal level with regards to higher FP FSH [8.85 ±13.51 vs. 4.55 ± 3.22; p0.031], higher testosterone [4.10 ± 12.36 vs. 0.31 ± 0.30; p0.032], lower FP estradiol [143.00 ± 132.94 vs. 206.09 ± 77.01; p0.004], lower LP progesterone [6.51 ± 9.04 vs. 12.08 ± 12.46; p0.009], and lower LP estradiol [120.77 ± 112.06 vs. 218.73 ± 103.06; p0.000] (see Table 4).

**Table 4:** Showing the relationship between medication and depression.

**Variables**	**CBZ*N =* 54**	**LEV*N =* 57**	**Control*N =* 50**	***F* test**	***p***	**p2**	**p3**
FP FSH (mIU/ml)	12.19 ± 16.40	8.85 ± 13.51	4.55 ± 3.22	4.798	0.242	**0.002** ^*^	**0.031** ^*^
FP LH (mIU/ml)	12.17 ± 12.91	13.33 ± 25.30	7.97 ± 18.35	1.070	0.765	0.177	0.219
FP progesterone (ng/mL)	7.98 ± 17.24	7.10 ± 10.70	15.20 ± 32.18	2.221	0.745	0.153	0.076
FP estradiol (pg/mL)	127.23 ± 154.93	143.00 ± 132.94	206.09 ± 77.01	5.459	0.565	**0.002** ^*^	**0.004** ^*^
FP PRL (ng/mL)	14.35 ± 24.25	18.30 ± 15.15	17.19 ± 7.430	0.746	0.314	0.429	0.640
LP FSH (mIU/ml)	11.29 ± 14.87	7.54 ± 9.35	13.20 ± 19.40	2.022	0.113	0.572	0.053
LP LH (mIU/ml)	12.80 ± 13.45	5.81 ± 4.18	7.27 ± 15.40	5.292	**0.000** ^*^	0.053	0.495
LP progesterone (ng/mL)	7.36 ± 16.87	6.51 ± 9.04	12.08 ± 12.46	2.715	0.741	0.110	**0.009** ^*^
LP estradiol (pg/mL)	234.74 ± 811.22	120.77 ± 112.06	218.73 ± 103.06	0.924	0.296	0.890	**0.000** ^*^
LP PRL (ng/mL)	14.39 ± 24.05	17.10 ± 9.38	17.67 ± 7.27	0.660	0.446	0.357	0.733
Testosterone (ng/dL)	2.42 ± 6.67	4.10 ± 12.36	0.31 ± 0.30	2.779	0.378	**0.027** ^*^	**0.032** ^*^

FP, follicular phase; LP, luteal phase; FSH, follicle stimulating hormone; LH, luteinizing hormone; PRL, prolactin; ^*^statistical significance, ^*^p1 is the p-value comparing carbamazepine and levetiracetam; ^*^p2 is the *p*-value comparing carbamazepine and controls; ^*^p3 is the *p*-value comparing levetiracetam and controls. Bold indicates significant values.

### 3.5 Linear regression showing predictors of depression among epilepsy patients

FP FSH, FP estradiol, FP PRL, LP progesterone, LP estradiol, LP PRL, and testosterone levels are associated with depression in patients with epilepsy. However, only LP PRL [B 0.038, std error 0.018, 95% CI 0.001–0.075; p0.042] and testosterone [B −0.806, std error 0.116, 95% CI −1.037 to −0.574; p0.000] predicted depression (see Table 5).

**Table 5:** Showing predictors of depression patients with epilepsy using linear regression.

**Variables**	**B**	**Std. error**	***p***	**95% CI** **Lower–upper bound**
FP FSH (mIU/mL)	−0.008	0.008	0.303	−0.024–0.008
FP estradiol (pg/mL)	−0.002	0.002	0.344	−0.005–0.002
FP PRL (ng/mL)	−0.029	0.020	0.156	−0.068–0.011
LP progesterone (ng/mL)	0.009	0.006	0.179	−0.004–0.022
LP Estradiol (pg/mL)	0.002	0.001	0.125	−0.001–0.005
LP PRL (ng/mL)	0.038	0.018	**0.042** ^*^	0.001–0.075
Testosterone (ng/dL)	−0.806	0.116	**0.000** ^*^	−1.037 to −0.574

FP, follicular phase; LP, luteal phase; FSH, follicle stimulating hormone; PRL, prolactin; ^*^statistical significance; B, beta; Std, standard; CI, confidence interval. Bold indicates significant values.

## 4 Discussion

This study revealed that the prevalence of depression among WWE was 18.8% when compared to previous studies carried out in Nigeria, which reported a prevalence ranging between 20.4% and 42% among PWE (Okubadejo et al., 2007; Gureje et al., 2010; Ogunrin et al., 2013; Sunmonu et al., 2019). Similarly, Onwuekwe et al. (2012) used the Becks Depression Inventory (BDI) and found a prevalence of 85.5% of depressive symptoms among PWE in South-East Nigeria. However, the values of prevalence vary from study to study, possibly due to different instruments used in assessing depression.

The mean ZSRDS depression in this study was higher in the WWE group than in the control group. Factors such as formal education and age group are known to be statistically significant with depression in epilepsy patients, and this was similar to what was observed in this study (Onwuekwe et al., 2012; Ojagbemi et al., 2022). An earlier study that evaluated the social and economic impact of epilepsy in PWE in Nigeria revealed that fewer years of education and lower marriages rates were more frequent among depressed compared to non-depressed in PWE (Sunmonu et al., 2008). Depression was common in the younger age (adolescent) of the age group 21–30 years, accounting for 10 (53.6%) out of a total of 21 patients with depression, which is similar to a report by Onwuekwe et al. (2012) on depression in PWE, which reports a higher prevalence in adolescents. The reason may be due to the onset of a longer duration of seizure in childhood nor early exposure to stigmatization. Female gender and epilepsy were documented as known risk factors of depression in previous studies. However, there are only a few studies conducted on sex hormones in women of different ages suffering from depression and epilepsy, and their conclusions are not uniform until now (Gureje et al., 2010; Gaus et al., 2015; Hamson et al., 2016).

This study attempted to unravel the knowledge gap between sex steroid hormones, epilepsy, and depression in WWE and found that changes in PRL and testosterone level are a potential biomarker of depression among PWE. However, the therapeutic implication of the above findings has not been fully explored. Thus, targeting levels of the sex steroid hormone might be a possible therapeutical approach against depression in WWE. For instance, alterations in signaling pathways and neuronal network function and sex steroid hormones play a major role in the pathophysiology of epilepsy in WWE and depression, and again, hormonal dysregulation due to the disruptive effects of seizures and interictal epileptiform discharges on the hypothalamic–pituitary–adrenal axis likely contributes to high rates of depression in epilepsy patients (Bhatta et al., 2018; Blair et al., 2019; Osalusi et al., 2020).

Early decline in ovarian function may be found with MDD patients. In a recent study on premenopausal women, Young et al. (2000) reported lower FP plasma estrogen levels and higher LH levels in women with depression than in control subjects. Since decreased estrogen influences a range of neuroendocrine systems related to mood, behavior, and cognition, it is conceivable that women with a history of depression may have a long-standing compromised hypothalamic–pituitary–ovarian axis. Early menarche is associated with development of depression later in life and also an earlier onset of menopause. Depression may, therefore, have either an intermediary or an independent effect on age at natural menopause. Thus, it is not surprising that the findings from this study showed that there was a lower hormone level in cases, except for FP FSH, FP LH, LP LH, and testosterone, which were higher than those in the controls.

Estrogen has long been suggested to have proconvulsant properties, but its effect might be more complex depending on the dose, route of administration, acute vs. chronic administration, natural hormonal environment, and estrogenic species. Progesterone has long been shown in several studies to have anti-seizure activities. The possible anti-seizure effect of progesterone could be mediated by its metabolite allopregnanolone; hence, the measurement of progesterone in plasma is essential to evaluate its efficacy in WWE who are prone to seizure in response to a decreased level of progesterone, especially during perimenstrual periods. Previous studies have not documented the effect of FSH on epilepsy; however, this study found higher FPFSH (Osalusi et al., 2020). Testosterone has a neuroprotective role and could enhance adult neurogenesis by increasing the survival of newly generated neurons with a minimal influence on cellular proliferation. There exists an inverse relationship between prolactin and dopamine levels. An increase in prolactin level is associated with a low level of dopamine and, consequently, higher signs and symptoms of depression (Harden and Pennell, 2013).

Progesterone has long been shown in several studies to have anti-seizure activities (Svalheim et al., 2008, 2009; Shiono et al., 2019). The possible anti-seizure effect of progesterone could be mediated by its active metabolite allopregnanolone; hence, the measurement of progesterone in plasma is mandatory to evaluate its efficacy in WWE who are rather prone to seizures in response to a decreased level of progesterone, especially during perimenstrual periods; this aligns with our study in which a lower progesterone level lower was observed in cases compared to controls. It has also been proposed that the incidence of epilepsy is lower in women than in men due to the effect of progesterone. Elevated estrogen or decreased progesterone levels can exacerbate seizure frequency. More specifically, progesterone or its metabolites (allopregnanolone) have been found to inhibit neuronal excitability through the modulation of the GABA type A receptor (Majewska et al., 1986). Estrogen is believed to promote seizure activity by enhancing the activation of the NMDA receptor in the hippocampus (Reddy et al., 2004). The most vulnerable time for catamenial epilepsy in an ovulating female is immediately prior to ovulation and prior to menses, when estrogen/progesterone ratios are high. In an ovulating female, catamenial epilepsy typically occurs during the latter half of the menstrual cycle due to unopposed elevated estrogen levels.

Previous studies have shown that the effect of ASM on depression is bidirectional and dependent on the mechanism of action of ASM (Kanner, 2005; Kanner et al., 2012; Adebayo et al., 2014; Ogundare et al., 2020). The implication of this is that not all ASMs are associated with depression. ASMs have a high psychotropic potential beyond their anti-seizure effect, which needs to be systematically investigated in epilepsy patients. However, it is well known that, especially in PWE, ASMs may have negative effects on mood and behavior. The ASMs most associated with the occurrence of depressive symptoms in PWE seem to be those which act at the benzodiazepine–GABA receptor complex and include barbiturates, topiramate, and vigabatrin (Mula and Sander, 2007).

Data on LEV show an intermediate risk and an incidence of depression of about 3% or lower. However, it is also known that ASMs like CBZ have positive effects, which include mood-stabilizing and antimanic effects and also antidepressant effects. However, the use of CBZ is limited due to the effects of drug–drug interaction involving the cytochrome p450; hence, the use of CBZ decreases the PRL level, in turn decreasing the prevalence of depression, while the use of LEV increases the PRL level. The exact mechanism of action of carbamazepine as a mood-regulating drug or an antidepressant is undetermined, but it works by reducing dopamine turnover, increasing GABA levels in the brain through a variety of synthesis and degradation processes, and modifying other neurotransmitters, voltage-sensitive Na^+^ channels, extrahypothalamic neuropeptides, secondary messenger systems, and providing neuroprotection (Ayano, 2016). Elevation in PRL inhibits the pulsatile release of LH and FSH, which inhibits the production of testosterone by the gonads. Gupta et al. (2017) concluded that plasma PRL was higher in individuals with MDD than in healthy controls and among women than in men, which is similar to the findings of our study. Historically, PRL levels recorded shortly after a seizure (within 20 mins) have been used to assess the etiology (epileptic or non-epileptic) of an epilepsy spell. Elevated serum PRL assay is a surrogate marker in epileptic seizure diagnosis.

Furthermore, this study showed that the FP is characterized by lower levels of hormones, except for FSH and LH, which were found to be higher in the CBZ group than in controls. In addition, the LP was characterized by lower levels of hormones, except for LH, estradiol, and testosterone, which were higher in the CBZ group than in controls. There was a higher hormonal level in the FP, except for the FSH, LH, and PRL, which were lower in LEV groups than in controls. There was a lower hormonal level in the LP, except for testosterone, which is higher in the LEV group than in controls. Although association between sex steroid hormones and depression exists, only PRL and testosterone predicted depression. This study is limited to adults only; thus, the effect of inflammation on depressive symptoms among young epilepsy patients could not be ascertained. Testosterone level was measured once in the study; thus, variation in testosterone level across different menstrual phases could not be ascertained. In addition, a majority of the cases recruited incidentally were on CBZ. As such, the effects of other AEDs on hormones and epilepsy could not be assessed.

## 5 Conclusion

The mean depression score was higher among cases when compared to controls, and there is a difference in hormonal levels between the two groups. Of all sex steroid hormones that are associated with epilepsy, only PRL and testosterone predicted depression in PWE. Clearly, changes in PRL and testosterone level are a significant biomarker of depression among PWE as the use of CBZ decreases the PRL level, which decreases the prevalence of depression. Therefore, the use of AEDs like CBZ with both mood-stabilizing and antimanic effects should be encouraged.

## Data availability statement

The raw data supporting the conclusions of this article will be made available by the authors, without undue reservation.

## Ethics statement

The studies involving humans were approved by Health Research Ethics Committee (HREC) of Olabisi Onabanjo University Teaching Hospital (OOUTH) Sagamu with assigned number OOUTH/HREC/275/2019AP and Federal Medical Center, Abeokuta (FMCA) with assigned number FMCA/243/HREC/03/2016/15. The studies were conducted in accordance with the local legislation and institutional requirements. The participants provided their written informed consent to participate in this study.

## Author contributions

LO: Conceptualization, Data curation, Formal analysis, Funding acquisition, Investigation, Methodology, Project administration, Resources, Software, Supervision, Validation, Visualization, Writing – original draft, Writing – review & editing. AAl: Data curation, Methodology, Supervision, Validation, Writing – original draft, Writing – review & editing. IO: Conceptualization, Data curation, Formal analysis, Funding acquisition, Investigation, Methodology, Project administration, Resources, Software, Supervision, Validation, Visualization, Writing – original draft, Writing – review & editing. JO: Data curation, Formal analysis, Methodology, Software, Writing – original draft, Writing – review & editing. EK: Data curation, Software, Validation, Visualization, Writing – original draft, Writing – review & editing. OOg: Data curation, Formal analysis, Methodology, Software, Writing – original draft, Writing – review & editing. OOl: Data curation, Formal analysis, Methodology, Writing – original draft, Writing – review & editing. PO: Data curation, Methodology, Writing – original draft, Writing – review & editing. OI: Data curation, Methodology, Writing – original draft, Writing – review & editing. AK: Data curation, Methodology, Writing – original draft, Writing – review & editing. SA: Data curation, Methodology, Writing – original draft, Writing – review & editing. MO: Data curation, Methodology, Writing – original draft, Writing – review & editing. DO: Conceptualization, Data curation, Formal analysis, Funding acquisition, Investigation, Methodology, Project administration, Resources, Software, Supervision, Validation, Visualization, Writing – original draft, Writing – review & editing. AAd: Data curation, Investigation, Methodology, Project administration, Software, Supervision, Writing – original draft, Writing – review & editing. EI: Data curation, Formal analysis, Investigation, Methodology, Software, Supervision, Writing – original draft, Writing – review & editing. AM: Data curation, Investigation, Methodology, Software, Supervision, Writing – original draft, Writing – review & editing. BO: Conceptualization, Data curation, Formal analysis, Funding acquisition, Investigation, Methodology, Project administration, Resources, Software, Supervision, Validation, Visualization, Writing – original draft, Writing – review & editing.
